# Retraction Note: Forensic characterization of 15 autosomal STRs in four populations from Xinjiang, China, and genetic relationships with neighboring populations

**DOI:** 10.1038/s41598-024-64860-5

**Published:** 2024-06-17

**Authors:** Xiaoni Zhan, Atif Adnan, Yuzhang Zhou, Amjad Khan, Kadirya Kasim, Dennis McNevin

**Affiliations:** 1 Department of Forensic Genetics, School of Forensic Medicine, China Medical University, Shenyang, 110122 P.R. China; 2 National Centre for Forensic Studies, Faculty of Science & Technology, University of Canberra, Canberra, Australia

Retraction of: *Scientific Reports* 10.1038/s41598-018-22975-6, published online 16 March 2018

The Editors have retracted this Article.

After publication concerns were raised about the consent process for participants in this study. Subsequent information provided by the Authors has indicated that a police officer may have been present as part of the team collecting blood samples and the Editors have concerns that this may have compromised the freedom of participants to give their consent to participate voluntarily.

Dennis McNevin and Atif Adnan disagree with this retraction. Xiaoni Zhan, Amjad Khan and Kadirya Kasim have not responded to correspondence from the Editors about this retraction. The Editors were not able to obtain a current email address for Yuzhang Zhou.

